# High-fidelity target sequencing of individual molecules identified using barcode sequences: *de novo* detection and absolute quantitation of mutations in plasma cell-free DNA from cancer patients

**DOI:** 10.1093/dnares/dsv010

**Published:** 2015-06-29

**Authors:** Yoji Kukita, Ryo Matoba, Junji Uchida, Takuya Hamakawa, Yuichiro Doki, Fumio Imamura, Kikuya Kato

**Affiliations:** 1 Department of Molecular and Medical Genetics, Research Institute, Osaka Medical Center for Cancer and Cardiovascular Diseases, Osaka 537-8511, Japan; 2 DNA Chip Research Inc., Yokohama 230-0045, Japan; 3 Department of Thoracic Oncology, Osaka Medical Center for Cancer and Cardiovascular Diseases, Osaka 537-8511, Japan; 4 Department of Gastroenterological Surgery, Osaka University Graduate School of Medicine, Osaka 565-0871, Japan

**Keywords:** massively parallel DNA sequencer, circulating tumour DNA, barcode sequences

## Abstract

Circulating tumour DNA (ctDNA) is an emerging field of cancer research. However, current ctDNA analysis is usually restricted to one or a few mutation sites due to technical limitations. In the case of massively parallel DNA sequencers, the number of false positives caused by a high read error rate is a major problem. In addition, the final sequence reads do not represent the original DNA population due to the global amplification step during the template preparation. We established a high-fidelity target sequencing system of individual molecules identified in plasma cell-free DNA using barcode sequences; this system consists of the following two steps. (i) A novel target sequencing method that adds barcode sequences by adaptor ligation. This method uses linear amplification to eliminate the errors introduced during the early cycles of polymerase chain reaction. (ii) The monitoring and removal of erroneous barcode tags. This process involves the identification of individual molecules that have been sequenced and for which the number of mutations have been absolute quantitated. Using plasma cell-free DNA from patients with gastric or lung cancer, we demonstrated that the system achieved near complete elimination of false positives and enabled *de novo* detection and absolute quantitation of mutations in plasma cell-free DNA.

## 1. Introduction

Circulating tumour DNA (ctDNA), which is the cell-free DNA (cfDNA) released from dying cancer cells to the blood, is an emerging topic in cancer research. Several proof-of-concept studies demonstrated that ctDNA could be used as a biomarker to monitor tumour burdens^1^ or acquired drug resistance.^2–4^ From a biological viewpoint, ctDNA is regarded as a carrier that brings the genetic information of solid tumours to peripheral blood. The use of ctDNA is expected to facilitate the analysis of genetic tumour heterogeneity,^5^ such as the evolution of cancer cells during the disease course, because of the difficulty in sampling recurrent or metastatic tissues. On average, cfDNA is fragmented to a size of 170 bp,^6^ and its half-life is estimated to be 16.5 min.^7^ One millilitre of blood contains the cfDNA from one to several thousand genomes. The rare mutations resulting from cancer cells must be detected in the vast amount of DNA from normal cells and quantitated.

Although various techniques have been used to detect ctDNA, digital polymerase chain reaction (PCR)^8^ and related technologies, particularly massively parallel DNA sequencers, are becoming the method of choice. In the case of massively parallel sequencers, the high read error rate of the current technologies is a major problem. Sequencing multiple sites or genomic regions dramatically increases the number of false positives, which is one of the reasons why current ctDNA assays tend to restrict their target to one or a few mutation sites.^6,9,10^ Another problem is the global amplification step during the template preparation for massively parallel sequencing. The final sequence reads do not represent the original DNA population, and the number of reads usually exceeds the number of target DNA molecules. Consequently, the quantitation of mutation alleles may be affected.

These problems can be solved using barcode sequences.^11,12^ Labelling DNA fragments with barcode sequences, typically N_10–15_, provides discrimination between the reads from individual molecules, thus enabling grouping of the reads from each molecule. Constructing a consensus of reads leads to both high-fidelity DNA sequencing and the capability to count the number of sequenced molecules. The main problem is the read errors that are introduced into barcode sequences, which can affect the basic principle of labelling each molecule with a single unique barcode. This problem has been recognized, and small collections of barcode sequences have been designed to detect and exclude erroneous sequences.^13,14^ However, this approach requires each barcode sequence to be individually manufactured. Therefore, it cannot accommodate large numbers of sequences.

As a first attempt, we focused on cancer-related genes that are frequently mutated, that is *TP53* and *KRAS*, and we developed a system for *de novo* detection and absolute quantitation of mutations in plasma cfDNA. This system consists of the following two steps:

- A novel target sequencing method that adds barcode sequences by adaptor ligation. This method uses linear amplification to eliminate the errors introduced during the early cycles of PCR.
- The monitoring and removal of erroneous barcode tags. This process involves the identification of individual molecules that have been sequenced and for which the number of mutations has been absolutely quantitated.

We named the collection of final consensus reads as ‘non-overlapping integrated reads (NOIR)’. The performance of the sequencing system, named the NOIR sequencing system, was demonstrated using plasma cfDNA from patients with gastric or lung cancer.

## 2. Materials and methods

### 2.1. DNA samples

We used Megapool Reference male DNA (Kreatech Biotechnology, Amsterdam, Netherlands), which is a pool of DNA from 100 normal Caucasian males, for technical development. The genomic DNA of leucocytes from normal individuals and the MIA PaCa-2 pancreatic carcinoma cell line, which has an R280W mutation in the *TP53* gene,^15^ was extracted using a standard phenol/chloroform protocol. Patients with activating *EGFR* mutations in lung cancer tissues were recruited at Osaka Medical Center for Cancer and Cardiovascular Diseases.^16^ A gastric cancer patient was recruited at Osaka University Hospital. Written informed consent was obtained from all patients recruited to this study. This study was approved by the ethics committee of Osaka Medical Center for Cancer and Cardiovascular Diseases and Osaka University Hospital.

Plasma was prepared via centrifugation of 4–5 ml of EDTA-treated blood at 800 *g* for 10 min at room temperature. The plasma was transferred to a fresh tube and re-centrifuged at 15,100 *g* for 10 min at room temperature. After centrifugation, the upper plasma was transferred to a fresh tube. The centrifuged liquid samples were frozen at −80°C until DNA extraction. DNA was extracted from 1.5 to 2.0 ml of a liquid sample using the QIAamp circulating nucleic acid kit (Qiagen, Hilden, Germany) according to the manufacturer's instructions. For several samples, the DNA concentration was determined using the Qubit dsDNA HS Assay Kit (Life Technologies, CA, USA).

### 2.2. Target regions, adaptors and region-specific primers

We designed adaptors and primers to analyse the genomic regions that code for the DNA-binding domain of *TP53* and for the mutation hotspots of *KRAS* and *CTNNB1* (Supplementary Tables S1–S3).

### 2.3. Library construction with linear amplification of the barcoded strands

Genomic DNA (5–40 ng) or cfDNA (from ∼1 ml of whole blood) was digested using multiple restriction enzymes [Set1: AlwNI and Alw26I; Set2: EarI and NcoI; SetKC: EarI and NmuCI (FastDigest enzymes, Thermo Scientific, MA, USA); Supplementary Table S1]. The ligation of adaptors with N_12_ barcode sequence tags was performed using *Escherichia coli* DNA ligase (Takara Bio, Shiga, Japan). The ligation products were purified twice with a 1.2× volume of AMPureXP beads (Beckman Coulter, CA, USA). Linear amplification of the purified products was performed with a region-specific primer mixture (Supplementary Tables S2 and S3) and Q5 Hot Start High-Fidelity DNA Polymerase (NEB) by 10 thermal cycles. The purified linear amplification products were amplified with the PGM/Proton primers (Supplementary Table S2) and Platinum Taq High Fidelity (Life Technologies). The amplification products were purified with AMPureXP beads or agarose gel electrophoresis with a MinElute Gel Extraction Kit (Qiagen). Further details and library construction for experiments with double strand labeling are provided in Supplementary Methods.

### 2.4. Massively parallel sequencing

For the Ion Torrent sequencing system, we prepared sequencing templates (emulsion PCR and bead-enrichment) from sequencing libraries using an Ion PI Template OT2 200 Kit v2 or v3 (Life Technologies) and an Ion OneTouch system (Ion OneTouch Instrument and Ion OneTouch ES, Life Technologies) according to the manufacturer's protocol. Prepared templates were sequenced using an Ion PI Sequencing 200 Kit v2 or v3 and the Proton sequencer (Life Technologies). Torrent Suite 4.0 or 4.2 (Life Technologies) was used to convert the raw signals into base calls and to extract the FASTQ files of the sequencing reads. Sequencing data from the Illumina system were generated using a MiSeq system (Illumina, CA, USA) according to the manufacturer's protocol, and the FASTQ files of single-end reads were extracted.

### 2.5. Data analysis

Reads in FASTQ format were divided using 5-bp indices for individual assignments. Sequences between the 5-bp indexes and spacer sequences were obtained as barcode tags. When the total length of the spacer and the following sequence was >70 bases, the reads were aligned to target sequences (spacer + target region) with bwa (version 0.6.2) using the bwasw mode for aligning long reads^17^ and the parameter setting ‘-b5 -q2 -r1 -z10’. Reads with long unmapped ends (>10% of the total read length) were discarded.

Barcode tags from mapped reads were analysed in each target region. Although we designed 12-bp barcode tags, we obtained tags that were not 12-bp in length due to insertion/deletion errors that were detected during sequencing. We discarded tags that were <9 bp. To recover the maximum number of reads, 11- and 13-bp tags that only differed from a 12-bp tag by the insertion or deletion of a single base were grouped with the corresponding 12-bp tag. For example, ‘TGCATGATACG’ and ‘TGCATGGATTACG’ were combined with the barcode group ‘TGCATGATTACG’.

Reads with the same barcode sequences were grouped together, and the barcode tags were assigned into 2-read bins according to the number of reads per tag. Then, the proportion of 12-bp tags in each bin was calculated, and the value (proportion) for each bin was averaged using the 11 bins around that bin. We defined the minimum bin as that with an average proportion of 90% or greater, and we set this value as the threshold for removing erroneous barcode tags. These processes were performed using an in-house Perl script.

After the removal of erroneous tags that had fewer reads than the threshold, the reads from tags with the same barcode were combined using samtools (version 0.1.18),^18^ and consensus sequences were created using VarScan (v2.2.11).^19^ If >50 reads were obtained, the longest 50 reads were analysed. If >80% of reads had an alternative base at a position, we assigned a variant. A set of consensus sequences was converted to a FASTQ file, and we assigned ‘57’ as a quality score for all bases. A FASTQ file was aligned to the sequences of target regions as described above, and the generated mapping data were processed to obtain the per base coverage (pileup files) using samtools. Subsequently, we summarized the base counts for each base position.

The sequence error rate was calculated by dividing the number of non-reference sequenced bases by the number of all sequenced bases in the target regions. When using barcode tags, consensus sequences of multiple reads from individual molecules were analysed. Reads before constructing consensus were used for deep sequencing. A common SNP site (dbSNP number: rs1800372) was not considered for calculation.

## 3. Results

### 3.1. Target sequencing method that adds barcode sequences by adaptor ligation

Barcode sequences can be attached to genomic DNA and transcriptomes by adaptor ligation.^11^ For target or amplicon sequencing, barcode sequences are embedded in PCR primers.^12^ Schmitt et al.^20^ proposed a method to label both strands of a sequence with the same barcode sequence to detect the base changes that occur in only one strand. This method can only be used for adaptors, and it cannot be applied to barcode primers.

**Figure 1.**
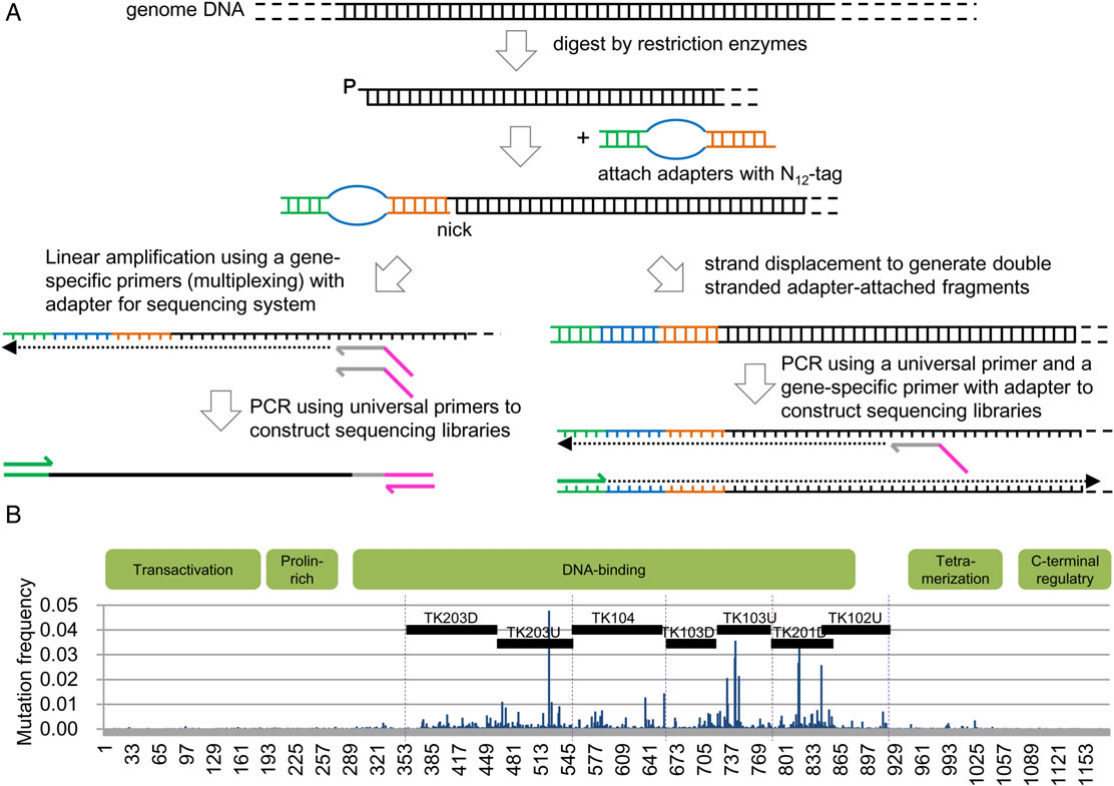
Construction of barcoded libraries. (A) Schematic of library construction. (B) Target regions in human *TP53*. The cDNA structure of the coding region of the human *TP53* gene is shown via a bar graph of the distribution of mutations found in COSMIC v63 (http://cancer.sanger.ac.uk/). The dotted lines indicate the boundaries of exons in the DNA-binding domain. The black bars indicate target regions defined in this study.

The addition of an adaptor to a restriction enzyme site and the subsequent PCR amplification with an adaptor-primer and a single gene-specific primer constitute a robust technique that our group has extensively applied to genomic DNA^21^ and RNA.^22,23^ We have used this method for target sequencing with barcodes. Restriction enzymes with five-, four- or three-base protruding ends can be used with this method and are listed in Supplementary Table S5. The listed collection of enzymes covers most of the human genome. We used *E. coli* DNA ligase, which enables the sequence-specific ligation of cohesive ends generated by type IIS restriction enzymes.^24^ The adaptor sequence that was used includes 5 bases for indexing individuals and N_12_ for indexing molecules (distinguishing up to 1.7 × 10^7^ molecules). We used two versions of the method (Fig. 1A). The first version involves linear amplification of the barcoded strand and subsequent PCR amplification. Linear amplification is expected to minimize errors in the first round of PCR. The second version employs replacement synthesis of the complementary strand to label both strands with the same barcode. For subsequent analysis, we primarily used the first version of the method with an Ion Proton sequencer. We chose the DNA-binding domain of *TP53*, which is covered by seven regions, as a target sequence (Fig. 1B and Supplementary Table S1).

### 3.2. Monitoring and removing erroneous barcode tags in the Ion Torrent system

**Figure 2.**
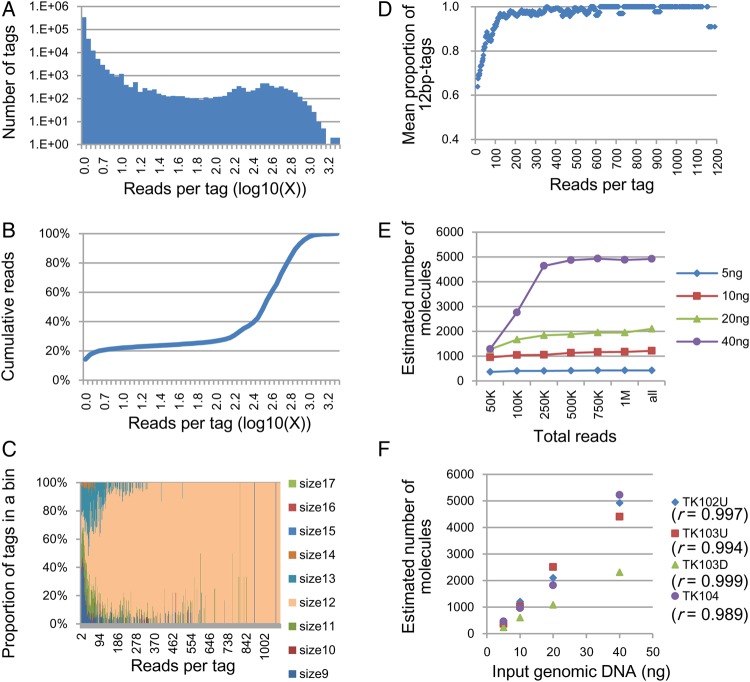
Monitoring the errors in the barcode sequence tags and absolute quantitation of target molecules. Results from Ion Proton sequencer data. The target region is TK102U, except in F. (A–D); results obtained using 40 ng of genomic DNA. (A) Distribution of reads per barcode tag. Vertical axis: number of different barcode tags. Horizontal axis: number of reads per tag, given as the common logarithm of the number. (B) Cumulative reads. (C) Proportions of barcode tags by size. (D) Mean proportion of 12-bp barcode tags after including the 11-bp and 13-bp tags that only differed from a 12-bp tag by the insertion or deletion of a single base. The mean proportion represents the average of the proportions of the nearest 11 bins. (E) Estimated number of target molecules after the removal of erroneous tags. Horizontal axis: number of reads used for estimation; the reads were randomly selected from the entire population of reads (‘5 ng’: 1,457,760; ‘10 ng’: 2,251,133; ‘20 ng’: 2,245,038; and ‘40 ng’: 2,395,763 reads). (F) Correlation between the number of molecules and the amount of input DNA after removal of the erroneous tags.

We sequenced four of the seven regions using genomic DNA with masses ranging from 5 to 40 ng. An example of the relationship between the number of barcode sequence tags and the number of reads grouped by the same barcode sequence tag (reads per tag) is shown in Fig. 2A. In this experiment, the input DNA corresponded to ∼10,000 copies of the genome, but the total number of tags was >400,000. The majority of these tags had a small number of reads, including single reads. However, the corresponding number of reads occupied only a small fraction of the total reads obtained (Fig. 2B). This phenomenon was observed in previous studies.^11,12,25^

Insertion/deletion errors occupy the majority (>90%) of sequencing errors in the output sequences from the Ion Torrent PGM/Proton.^26,27^ Consequently, the tags generated by read errors can be monitored using the tag size. The observed fractions of tags were grouped by size and are shown in Fig. 2C. Non-12-bp tags, that is erroneous tags, occupied the majority of tags with low numbers of reads per tag, and the fraction of 12-bp tags gradually increased as the number of reads per tag increased. This dynamic suggests that erroneous tags accumulate in the low read number fraction and can be removed by setting an appropriate threshold. To recover the maximum number of reads, we grouped the 11-bp and 13-bp tags that matched 12-bp tags with the exception of a single inserted or deleted base with their matching 12-bp tags. Then, we plotted the fraction of 12-bp tags against the number of reads (Fig. 2D). The minimum bin, where the average of the proportion of 12-bp tags in the nearest 11 bins was >90%, was defined as the threshold for removing erroneous tags. The fraction of 12-bp tags obtained exceeded 95% of the recovered fraction, and little improvement was observed when using more stringent thresholds. This threshold successfully separated the two peaks observed in Supplementary Figure S2A. The chosen thresholds varied depending on factors such as the total number of reads and the targeted regions: from 11 to 249 (data points of Fig. 2E) and from 57 to 485 (Fig. 2F). Through this process, 10–20% of the total reads were discarded (Supplementary Figure S2B).

We could not estimate the number of removed error-free tags by counting 12-bp tags, because the 12-bp tags in the fraction with a small number of reads contained sequences of the original size due to multiple insertion/deletion errors. Because the right peak (Fig. 2A) represented the peak of the distribution of the error-free tags, we removed the fraction corresponding to the tail of the distribution. When *M* is defined as the threshold value, the number of error-free tags between 0 and *M* should not exceed the number of tags between *M* and 2*M. From the number of 12-bp tags between *M* and 2*M, the estimated maximum for the removed error-free tags was 5–10% of the total error-free tags.

The number of targeted molecules can be measured using exhaustive sequencing. The number of tags obtained reached saturation at 500,000 reads (Fig. 2E). A linear relationship between the estimated number of target molecules and the amount of input DNA was observed, and the correlation coefficient was calculated to be >0.98 (Fig. 2F). Approximately 40% of the input DNA was recovered, except for TK103D, which exhibited 15% recovery. This calculation was based on the number of sequenced molecules. The difference in ligation efficiency among ligation sites is a likely cause of recovery variation.

There was a good linearity between the mutant-to-normal allele ratios of the artificial DNA mixtures and those deduced using the above procedure (Supplementary Figure S3).

Previous studies employed arbitrary criteria for removing tags with a small number of reads, such as the removal of tags with a single read. After applying a criterion to remove 1- or 2-read tags, a considerable fraction of erroneous tags remained, and the number of tags exceeded the number of target molecules estimated from the amount of input DNA. The number of tags increased with the addition of reads and did not reach saturation (Supplementary Figure S4), suggesting the generation of new erroneous tags.

### 3.3. Monitoring and removing erroneous barcode tags in the Illumina system

**Figure 3.**
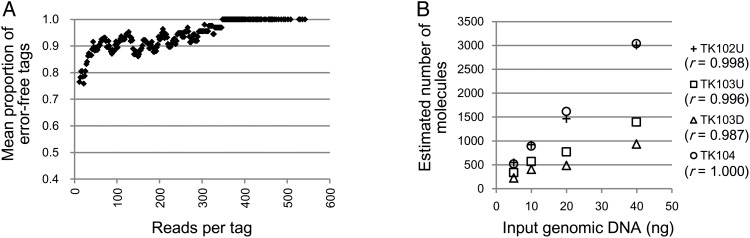
Monitoring the errors in the barcode sequence tags and absolute quantitation of target molecules. Results from MiSeq sequencer data. (A) Estimated mean proportion of error-free barcode tags. The mean proportion was calculated as in Fig. 2D. (B) Correlation between the number of molecules and the amount of input DNA after removal of the erroneous tags.

The read error profile of Illumina sequencers is different from that of Ion Torrent PGM/Proton sequencers. When using Illumina systems, substitution errors dominate.^26,27^ However, the observed distribution pattern of barcode tags was the same with both sequencing systems (Supplementary Figure S5A). To accommodate Illumina sequencers, we used ‘BDHVBDHVBDHVBDH’ as an error-detecting barcode. Here, each base position lacks one of the four bases, and the appearance of that missing base indicates a read error. We can estimate the total number of erroneous tags by multiplying the number of tags with missing bases by three (Fig. 3A, Supplementary Figure S5C). We then determined the threshold for removing erroneous tags from the distribution of error-free tags, as described above. The results obtained with Illumina sequencers were similar to those obtained using Ion Proton sequencers: 10% of the reads were removed (Supplementary Figure S5B); the values of the threshold varied from 15 to 65 (data points of Fig. 3B); the number of tags was saturated with exhaustive sequencing (Supplementary Figure S5D), but it increased continuously with the use of an arbitrary threshold (Supplementary Figure S5E); and linearity was observed between the estimated number of target molecules and the amount of input DNA (Fig. 3B).

### 3.4. Increased accuracy from building a consensus of reads from individual molecules

**Figure 4.**
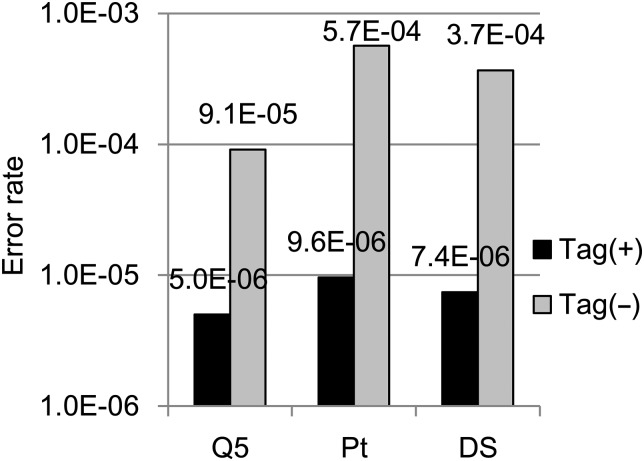
Sequencing error rates for the target regions. Substitution error rates with (black) and without (gray) barcode tags. Q5, single strand labelling with Q5 DNA polymerase for PCR amplification; Pt, single strand labelling with the Platinum Taq DNA polymerase High Fidelity kit for PCR amplification; DS, double strand labelling. Thirty nanograms of genomic DNA were used. The calculations were based on the sequence data from the seven (Q5, Pt) or five (except TK102 and TK103U for DS) regions obtained using an Ion Proton sequencer. Ninety-five per cent confidence intervals of the error rates are as follows: Q5 tag+, 2.8 × 10^−6^– 8.8 × 10^−6^; Pt tag+, 6.9 × 10^−6^– 1.3 × 10^−5^; DS tag+, 3.3 × 10^−6^– 1.6 × 10^−5^; Q5 tag−, 9.0 × 10^−5^– 9.3 × 10^−5^; Pt tag−, 5.7 × 10^−4^–5.7 × 10^−4^; DS tag−, 3.7 × 10^−4^– 3.7 × 10^−4^.

The use of barcode tags enables high-fidelity sequencing by grouping and constructing a consensus of multiple sequences generated from a single molecule. The accuracy of this method is shown in Fig. 4 and Supplementary Figure S6. The error rates using barcode tags overlapped with a 95% confidence interval due to the small number of non-reference bases. Although we compared the results of using DNA polymerases with different proofreading functions (Q5 DNA polymerase from NEB and Platinum Taq DNA polymerase High Fidelity from Life Technologies) for the PCR amplification step of library construction, there was no significant difference when using barcode tags (Fig. 4). The second version of the barcoding method, which involved labelling both strands with the same barcode sequence, did not improve the accuracy compared with that of the single strand labelling (Fig. 4). For the first version, we used linear amplification cycles prior to PCR, which minimized the errors obtained in the early PCR cycles. This may explain why the second version of the barcoding method did not offer advantages over the single strand labelling method.

The accuracy of the first version of the barcoding method using the Illumina system was 1.8 × 10^−6^ (95% confidence interval, 3.5 × 10^−8^– 6.9 × 10^−6^).

### 3.5. Analysis of plasma cfDNA from a gastric cancer patient

**Figure 5.**
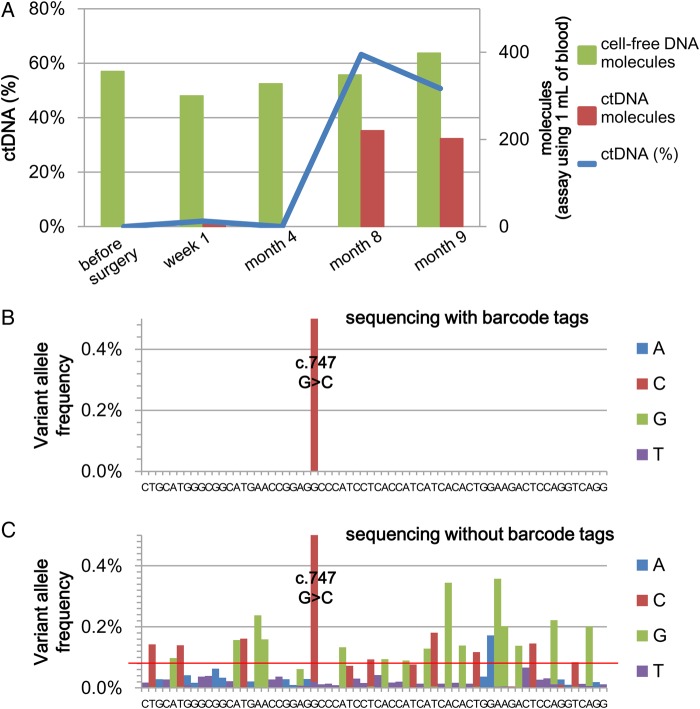
Dynamics of ctDNA during the disease progression of a gastric cancer patient revealed using the NOIR sequencing system. (A) The levels of the *TP53* mutant fraction in the cell-free DNA, that is ctDNA (%), and the number of total and mutation-positive molecules at various time points after the patient was operated on are displayed. (B and C) Fraction of calls not matching the reference sequence (Month 9): NOIR (B); deep sequencing, 1.7 total million reads (C). The red line indicates the criterion for variant detection proposed by Couraud et al.

We tested the NOIR sequencing system by monitoring the disease progression of a gastric cancer patient (73-yr-old male). The clinical characteristics of this patient were as follows: distal gastrectomy for a 65-mm tumour with multiple lymph node metastases; positive intraoperative peritoneal cytology; emergence of multiple liver metastases in spite of post-operative S-1 chemotherapy. This patient's primary tumour carried a mutation in *TP53* (c.747G > C), and we used TK103U (Fig. 1) to measure the level of the mutation at five different time points during the progression of the disease (Fig. 5A, Supplementary Table S6). The ctDNA level represented by the *TP53* mutation was zero or low during the early period of the disease and increased during the later period when the disease had progressed. In contrast, the level of cfDNA had no significant changes. Except for the above mutation, there were no base changes in any of the consensus reads.

We compared the above results with those obtained by deep sequencing using reads before the construction of NOIR (Fig. 5B and C). With the criterion of variant detection proposed by Couraud et al.,^28^ >20 positions were erroneously identified as variants.

### 3.6. Mutation screening in lung cancer

We performed a model screening experiment using cfDNA from lung cancer patients. The samples were originally collected for a clinical study of *EGFR* mutations, for which the statuses of the biopsy samples were available. We used *KRAS* (a 73-bp region) and *CTNNB1* (a 63-bp region) for the target genes. *KRAS* has a hotspot region in codons 12 and 13. In lung cancer, the *KRAS* and *EGFR* mutations are exclusive and rarely co-exist in the same patients. Unlike *KRAS* and *EGFR, CTNNB1* mutations are infrequent in lung cancer.^29^ *KRAS* hot spot mutations should appear only in *EGFR* mutation-negative lung cancer, and no mutations should be found in samples from normal individuals.

When the number of base changes in a target region is significantly higher than the average, we may attribute the changes to variant(s). We can use a statistical model to calculate the probability that a specific number of sequencing errors will occur. The average number of base changes due to sequencing errors, *λ*, is as follows:

λ=l×m×ER

where, *l*, *m* and ER are the number of base pairs in a target region, the number of sequenced molecules and the sequencing error rate, respectively. With the application of a Poisson distribution model for the sequencing error, the probability of *n* or more sequencing errors is as follows:

$$P=1-\overset{n-1}{\underset{k=0}{\sum }}\frac{\;{\left(l\times m\times \text{ER}\right)}^{k}{\text{e}}^{-l\times m\times \mathrm{ER}}}{k!}$$

In the following experiments, we set *P* = 10^−3^, that is one false positive in 1,000 samples, as the criterion for anomaly detection.^16^ Cases with *P* ≤ 10^−3^ are regarded as variant(s)-positive. ER was set to 10^−5^ for NOIR and 5 × 10^−4^ for deep sequencing. It should be noted that this criterion evaluates the entire target region and not individual base positions.

**Figure 6.**
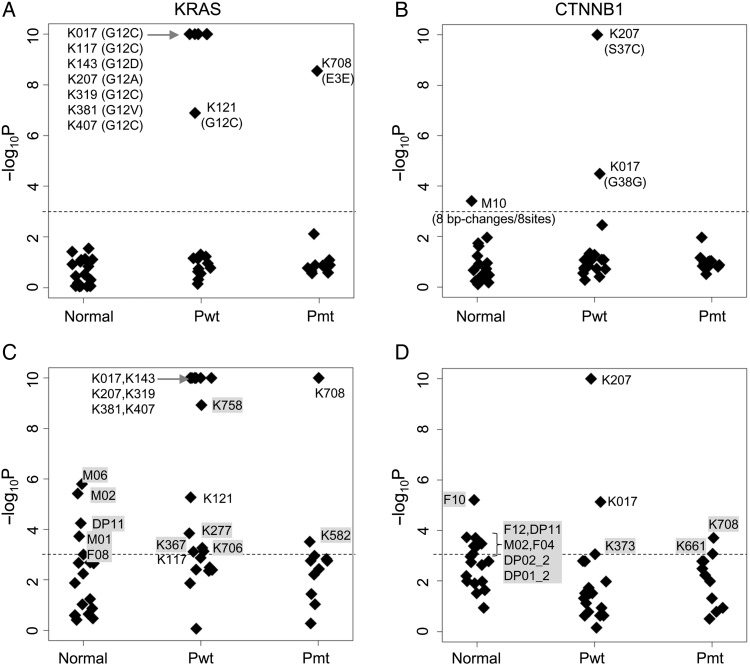
*De novo* detection of *KRAS* mutations in plasma cell-free DNA from lung cancer patients. The vertical axis indicates the *P*, that is the probability of false positives, described in the main text. Horizontal axis: normal, leucocyte or plasma cell-free DNA from normal individuals; Pwt, *EGFR* mutation-negative patients; Pmt, *EGFR* mutation-positive patients. The plots correspond to *KRAS* (A and C) and *CTNNB1* (B and D). A and B show the results from the NOIR sequencing system. C and D show the results from randomly extracted sequence reads (500 reads from plasma samples and 4,000 reads from leucocyte samples) without the use of barcodes. The dotted lines indicate the threshold for variant detection (*P* = 0.001). False-positive samples in C and D are marked with grey background.

The number of samples was 11 of *EGFR* mutation-positive lung cancer, 19 of *EGFR* mutation-negative lung cancer, 11 of leucocyte DNA from normal individuals and 7 of cfDNA from normal individuals. There was no significant loss of template molecules with these two genes during the assay process. The results are shown in Fig. 6A and B, and the corresponding data are provided in Supplementary Tables S7 and S8. Among the nine variant-positive cases in the *KRAS* experiment, eight were *EGFR* mutation-negative lung cancers that had substitutions in codon 12, and one was an *EGFR* mutation-positive case in which the substitutions were non-synonymous and located outside of the hotspot region. All cases with base changes in the hotspot region (codon 12) were judged as variant-positive. Among the three variant-positive cases in the *CTNNB1* experiment, two cases were *EGFR* mutation-negative, and the base changes were a non-synonymous mutation and a synonymous mutation. One variant-positive case showed normal leucocyte DNA; this is the sole false-positive case. Because redundancy in sequenced molecules was removed, we can discriminate experimental errors and true mutations from distribution patterns of base changes. Base changes in this case were regarded as experimental errors, because all eight base changes were located at different base positions. In contrast, base changes in all other variant-positive cases were accumulated at a single base position. The results agreed well with previous knowledge of the mutations. Because *KRAS* mutations have been found in 25% of lung cancers,^30^ the above results suggest that most of the *KRAS* mutation-positive lung cancers were likely to be detected using plasma cfDNA.

We repeated the experiment with randomly selected raw reads (reads before the consensus was built) as a model of deep sequencing^16^ (Fig. 6C and D). The *P*-value of five and seven normal samples in the KRAS and *CTNNB1* experiments was less than the threshold, and judged as variant-positive. For deep sequencing, less stringent threshold was required to eliminate these false positives.

## 4. Discussion

By using the NOIR sequencing system, we successfully eliminated false positives and could detect *de novo* and quantitate the mutations in plasma cfDNA. Therefore, it would be worthwhile to further evaluate the system in prospective clinical studies.

The current system is designed for *KRAS* and the hotspot region of *TP53*, which cover up to 50% of the cancer population, but the system can easily be extended to include other genes, such as *PI3KCA*, *PTEN* and *EGFR*. Our standard assay protocol uses plasma cfDNA from 1 ml of whole blood for a single reaction,^16^ and this system can process 2–4 DNA fragments that are ∼100 bp long in a single reaction. Thus, standard blood sampling (5–10 ml) can cover a genomic region of 1–4 kb. Application of multiplex nested PCR may increase the number of target genes beyond this limitation. Moreover, the principle may be applied to exome sequencing. It is important to note that the NOIR sequencing system still introduces sequencing errors. As the size of the target regions increases, the false positives will increase. Accurate evaluation of the probability of false positives is important.

In the NOIR sequencing system, the barcode technology plays the most important role. The principle for our absolute quantitation can be applied to other systems such as RNA-seq. Deakin et al.^31^ examined read errors within libraries of small numbers of barcode sequences. Their analysis revealed that the erroneous barcode tags localized in the fraction containing tags with small numbers of reads, and few of these tags were found in the fraction with large numbers of reads. Although the complexity of the barcode sequences was low, the results agreed well with those obtained in our study. Additionally, two or three barcode tags out of 100 were reported to be lost during PCR. The loss was likely sufficiently small to not introduce a bias that would affect the practical application of the barcode technology.

A possible alternative to the NOIR sequencing system is Safe-SeqS,^12^ which uses a barcode (unique identifier) for rare mutation detection. Its error rate is similar to ours, but a direct comparison may not be valid due to the different sequencing platforms and experimental conditions. The current version of Safe-SeqS uses two or four PCR cycles for barcode addition and does not satisfy the basic principle of labelling each molecule with a single unique barcode. Safe-SeqS does not measure the errors that are introduced into barcode sequences either. Thus, absolute quantitation is not possible, and the final consensus reads are redundant. This redundancy would reduce the statistical power of the mutation detection.

Other deep sequencing approaches had higher error rates. Background frequencies of non-reference reads in Tam-Seq were ∼0.1%,^32^ because using Q30 reads. Narayan et al.^33^ employed overlapping paired-end reads to reduce miscalls. They could reduce the miscall frequency to 0.014%, which was still significantly higher than those obtained with the barcode technologies.

ctDNA has two unique features that clinical parameters do not have. One is that ctDNA carries genetic information from malignant tumours, and this information can be obtained through peripheral blood sampling. Another feature is that ctDNA is released from dying cancer cells, and its level reflects tumour burden and/or effects of anti-cancer drugs. Absolute quantitation of ctDNA would provide accurate information on the ctDNA dynamics to guide patient treatment.

## 5. Data availability

The sequence data were deposited in the DDBJ Sequence Reads Archive [DDBJ:DRA002833].

## Supplementary data

Supplementary data are available at www.dnaresearch.oxfordjournals.org

## Funding

This work was partly supported by the Ministry of Education, Culture, Sports, Science and Technology, Japan [KAKENHI 25430180]. Funding to pay the Open Access publication charges for this article was provided by the Osaka Medical Center for Cancer and Cardiovascular Diseases.

## Supplementary Material

Supplementary Data
