# Supplementary material for: High-fidelity target sequencing of individual molecules identified using barcode sequences: de novo detection and absolute quantitation of mutations in plasma cell-free DNA from cancer patients
Source: DNA Res. 2015 Jun 29;22(4):269–77. doi: 10.1093/dnares/dsv010 (PMC4535617; doi:10.1093/dnares/dsv010)
Supplement: Supplementary Data [file supp_22_4_269__index.html]

High-fidelity target sequencing of individual molecules identified using barcode sequences: de novo detection and absolute quantitation of mutations in plasma cell-free DNA from cancer patients — Supplementary Data 

# High-fidelity target sequencing of individual molecules identified using barcode sequences: *de novo* detection and absolute quantitation of mutations in plasma cell-free DNA from cancer patients

## Supplementary Data

Supplementary Data

- Supplementary Data - Pdf file
