# Supplementary material for: High-fidelity target sequencing of individual molecules identified using barcode sequences: de novo detection and absolute quantitation of mutations in plasma cell-free DNA from cancer patients
Source: DNA Res. 2015 Jun 29;22(4):269–77. doi: 10.1093/dnares/dsv010 (PMC4535617; doi:10.1093/dnares/dsv010)
Supplement: Supplementary Data [file supp_dsv010_dsv010supp.pdf]

Supplementary Data

Supplementary Methods

Library construction with linear amplification of the barcoded strands

Genomic DNA (5-40 ng) or cell-free DNA (from ~1 mL of whole blood) was digested using multiple restriction enzymes [Set1: AlwNI and Alw26I; Set2: EarI and NcoI; SetKC: EarI and NmuCI (FastDigest enzymes, Thermo Scientific, MA, USA); Supplementary Table S1] in 12 µL of 1x FastDigest buffer with incubations of 10 min at 30 °C, 20 min at 37 °C, 10 min at 45 °C, and 20 min at 65 °C. The ligation of adaptors with N12 barcode sequence tags was performed in 24 µL of solution containing the restriction enzyme digestion, 12 nmol of NAD, 12 pmol of mixed N12-tag adaptors, and 60 units of E. coli DNA ligase (Takara Bio, Shiga, Japan) with four incubation cycles of 1 hr at 16 °C, 1 hr 13 °C, and 1 hr at 10 °C. After incubation, the reaction solutions were stored at 16 °C or -30 °C (for long storage periods) until the next step. The ligation products were purified twice with a 1.2x volume of AMPureXP beads (Beckman Coulter, CA, USA). The purified beads were dissolved in 20 µL of the linear amplification solution: 1x Q5 Reaction Buffer (NEB, MA, USA), 0.2 mM dNTPs, 2 µM region-specific primer mixture (Supplementary Tables S2 and S3), and 0.4 units of Q5 Hot Start High-Fidelity DNA Polymerase (NEB). After removal of the AMPureXP beads, amplification was performed as follows: 30 sec at 98 °C for denaturation and 10 cycles of 10 sec at 98 °C, 10 sec at 68 °C, and 30 sec at 72 °C. The linear amplification products were purified once with a 1.2x volume of AMPureXP, and the purified beads were dissolved in 20 µL of PCR amplification solution: 1x High Fidelity PCR Buffer (Life Technologies), 0.2 mM dNTPs, 2 mM $MgSO_4$, 0.5 µM each of the PGM/Proton primers (Supplementary Table S2), and 0.4 units of Platinum Taq High Fidelity (Life Technologies). Thermal cycling after the removal of the AMPureXP beads was performed as follows: 2 min at 95 °C for denaturation and 30 cycles of 15 sec at 95 °C and 1 min at 60 °C (for TP53) or 63 °C (for KRAS/CTNNB1). To compare error rates between the DNA polymerases that were used for the final amplification step, purified linear amplification products were also amplified in 20 µL of solution: 1x Q5 Reaction Buffer, 0.2 mM dNTPs, 0.5 µM PGM/Proton primers, and 0.4 units of Q5 Hot Start High-Fidelity DNA Polymerase. Thermal cycling was performed for 30 sec at 98 °C for denaturation and 30 cycles of 10 sec at 98 °C, 10 sec at 65 °C, and 15 sec at 72 °C. The final amplification products that were obtained using Platinum Taq High Fidelity exhibited clearer bands after agarose gel electrophoresis than those obtained using Q5 DNA polymerase; thus, we primarily used Platinum Taq High Fidelity for the final amplification. For the preparation of libraries for analysis by the Illumina system, the final PCR amplification step was performed

using indexed oligonucleotides for the discrimination of individual samples (Supplementary Table S3) and Platinum Taq High Fidelity. The amplification products were purified twice with a 1.0x volume of AMPureXP and eluted in 20 µL of nuclease-free water (Ambion, TX, USA). When the final amplification products were prepared using Q5 DNA polymerase, the suggested method for the Illumina system, or the KRAS/CTNNB1 assay, the products were purified using agarose gel electrophoresis with a MinElute Gel Extraction Kit (Qiagen).

Library construction for experiments with double strand labeling

The digestion of genomic DNA by restriction enzymes and adaptor-ligation were performed as described above. The purified ligation products were mixed in 20 µL of Platinum Taq High Fidelity PCR solution containing 0.5 µM T_PCR_A and a 0.5 µM region-specific primer mixture (Supplementary Table S2). The PCR mixture was incubated for 20 or 30 min at 72 °C for replacement synthesis by Pyrococcus GB-D polymerase using the Platinum Taq DNA Polymerase High Fidelity kit and amplified as follows: 30 cycles of 15 sec at 95 °C and 1 min at 60 °C. The validity of the double strand labeling was confirmed using a model experiment with a mixture of heteroduplex DNA fragments. PCR fragments from normal individuals (Megapool) and from a cell line with a mutation in TP53 (MIA PaCa-2) were mixed at a ratio of 100 to 1, denatured, and renatured. Library construction was performed as described above, with or without replacement synthesis. The use of replacement synthesis resulted in an approximately 10-fold reduction in the rate of mutation detection (Supplementary Figure S1 and Table S4).

Supplementary Figure S1

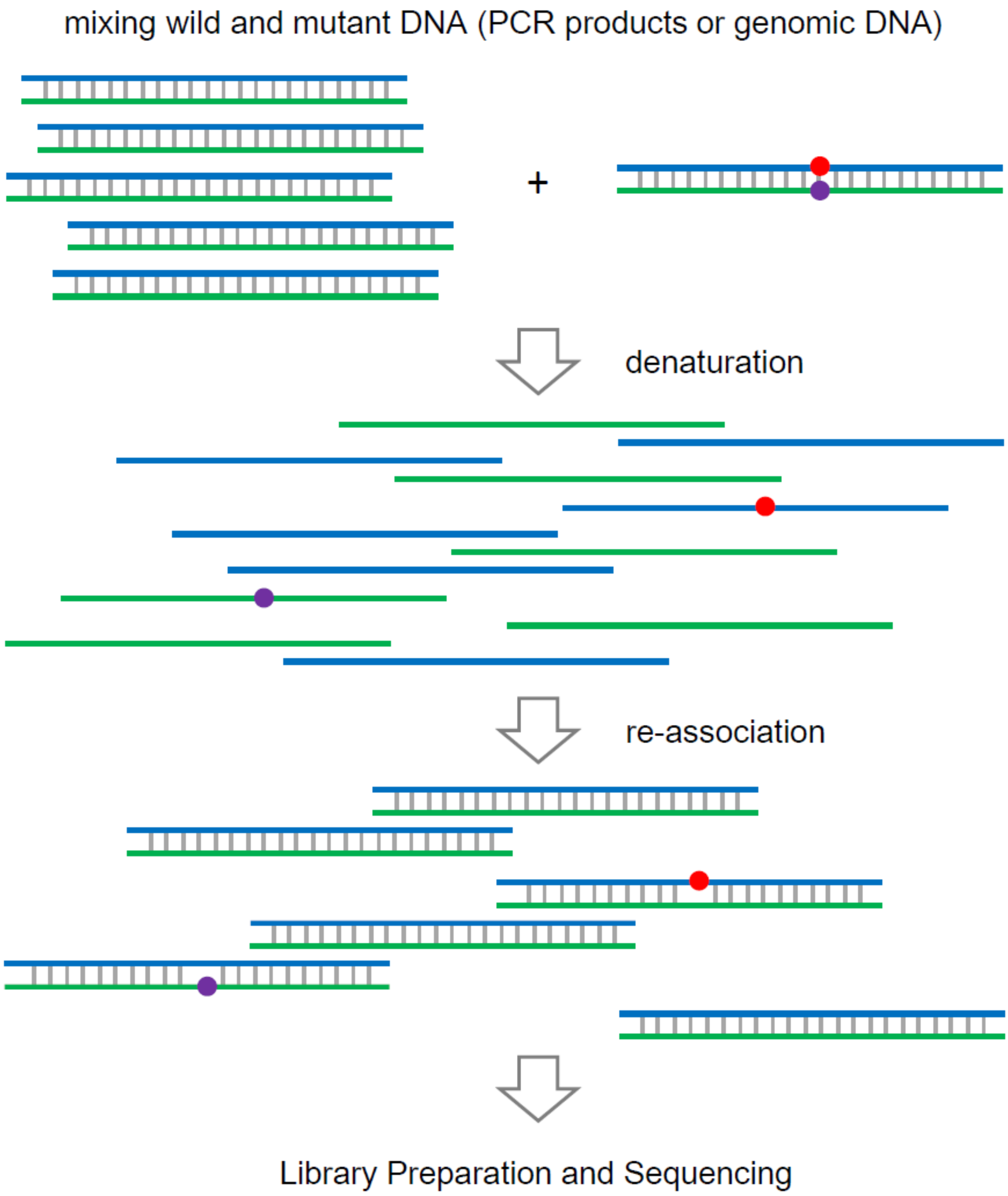

Supplementary Figure S1. Construction of heteroduplex fragments with base substitutions.

As models for DNA lesions, we prepared heteroduplex DNA fragments by mixing R280W mutant (from MIAPaCa-2) and wild type (from Megapool) PCR fragments at a ratio of 100 to 1. These artificial heteroduplex fragments were digested by restriction enzymes and attached to adaptors. We then generated fully double-stranded fragments using the strand displacement capability of Pyrococcus GB-D polymerase using a Platinum Taq DNA Polymerase High Fidelity kit (Life

STechnologies) and amplified the sequences by PCR without linear amplification (**Figure 1A**). The libraries were sequenced and analyzed using the barcode-tags.

Supplementary Figure S2

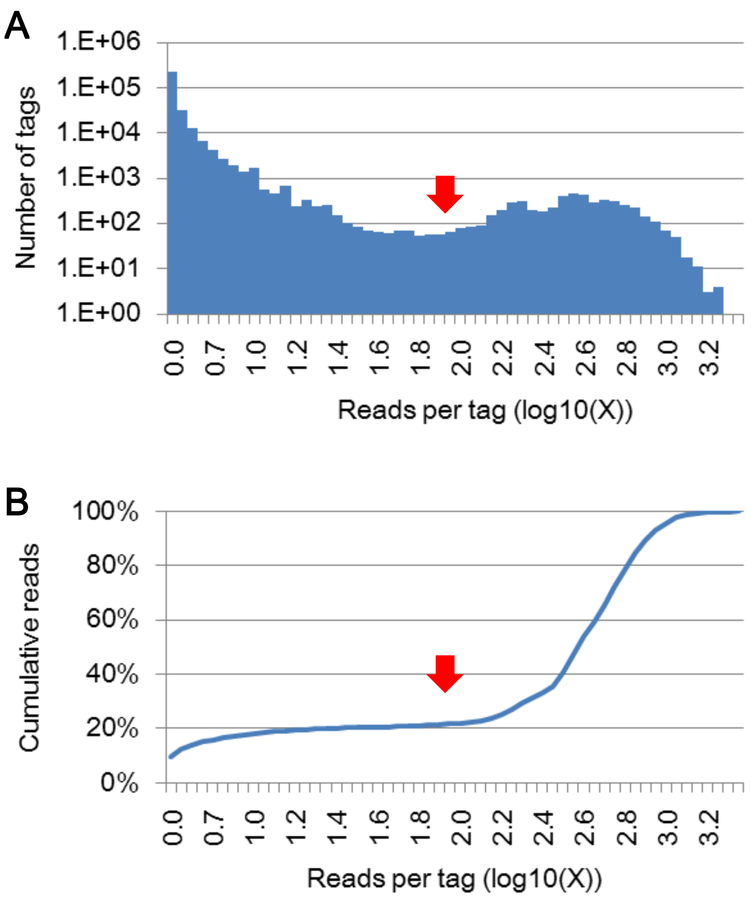

Supplementary Figure S2. Distribution of reads from the Proton sequencer after grouping 11-bp and 13-bp tags that matched 12-bp tags except for a single inserted or deleted base with those 12-bp tags. (**A**) Distribution of reads per barcode tag. Vertical axis: number of different barcode tags. Horizontal axis: number of reads per tag, shown as the common logarithm. (**B**) Cumulative reads. The arrow indicates the threshold for removing reads with erroneous tags. The analyzed region was TK102U. A total of 40 ng of genomic DNA was analyzed (2,395,763 reads).

Supplementary Figure S3

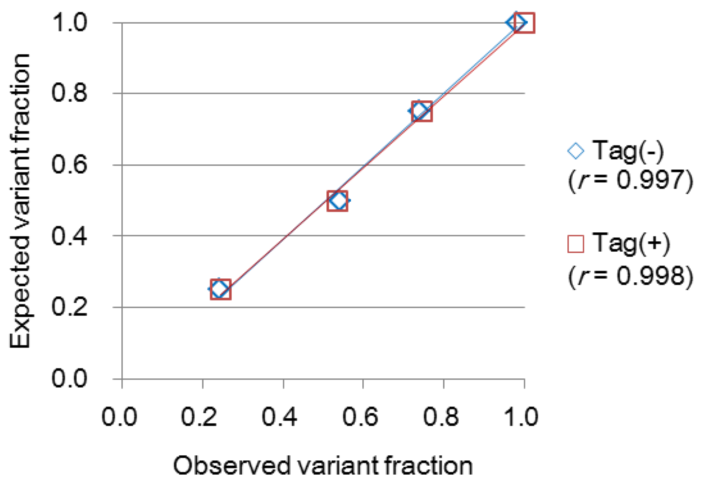

Supplementary Figure S3. Quantitative analysis. Genomic DNAs form MIA PaCa-2 with TP53 R248W homologous mutation and from Megapool (wild type) were mixed at ratios, 1:0, 3:1, 1:1, 1:3, and they were used for preparation of sequencing libraries as described in method section. Fractions of variants (R248W) were analyzed with the use of barcodes (Tag(+)) or without that (Tag(-)).

Supplementary Figure S4

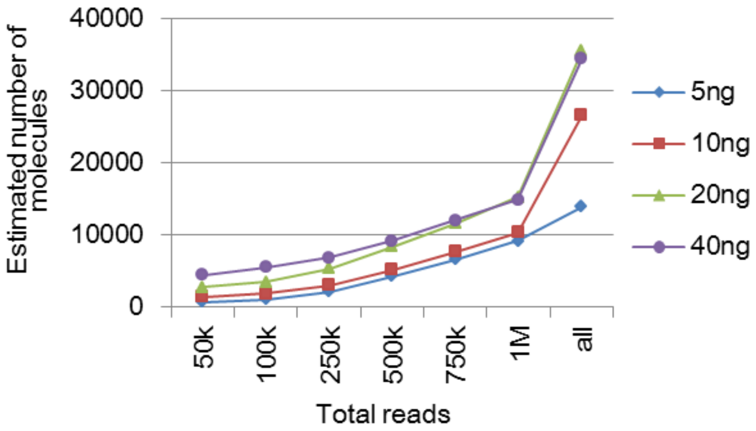

Supplementary Figure S4. Estimated number of target molecules after removing 1- or 2-read tags. The data used in **Figure 2E** were reanalyzed.

Supplementary Figure S5

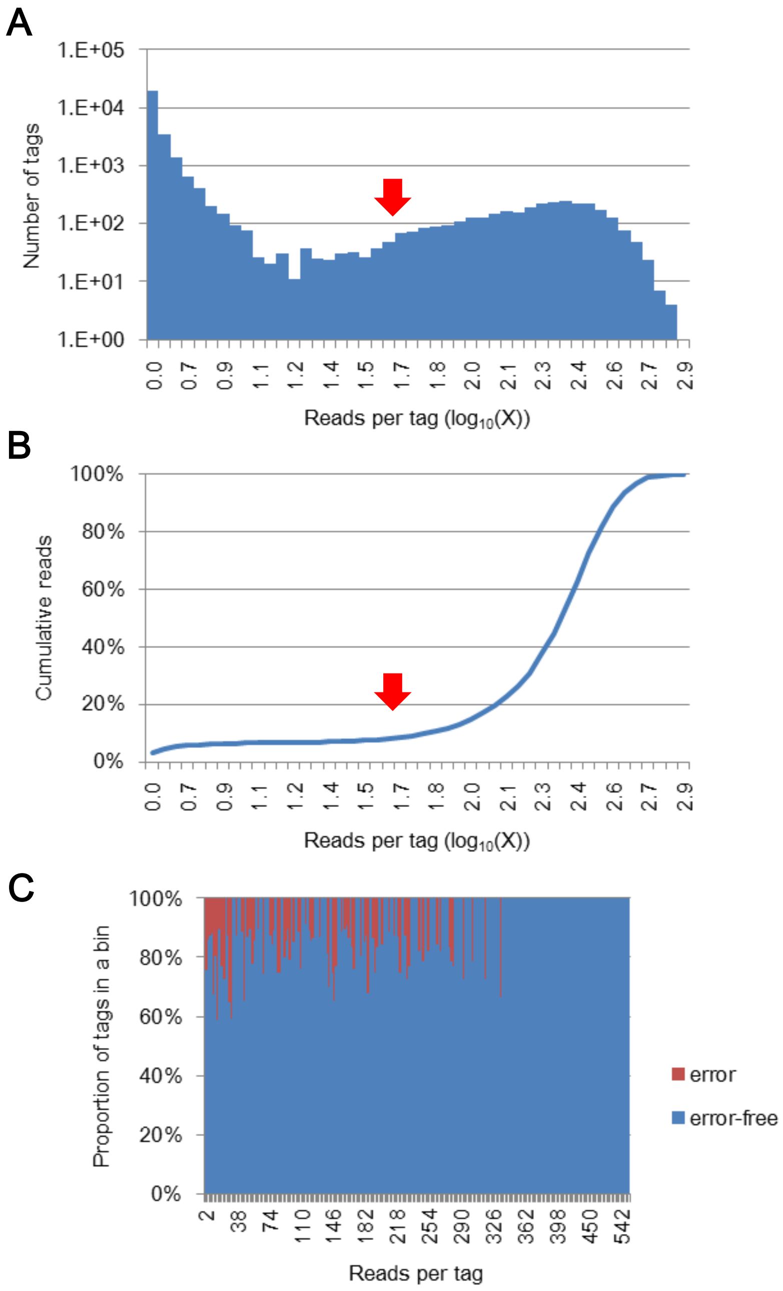

D

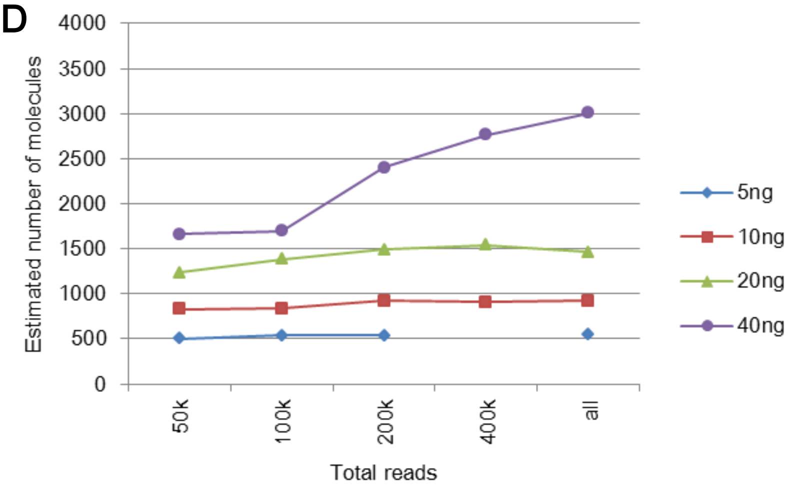

E

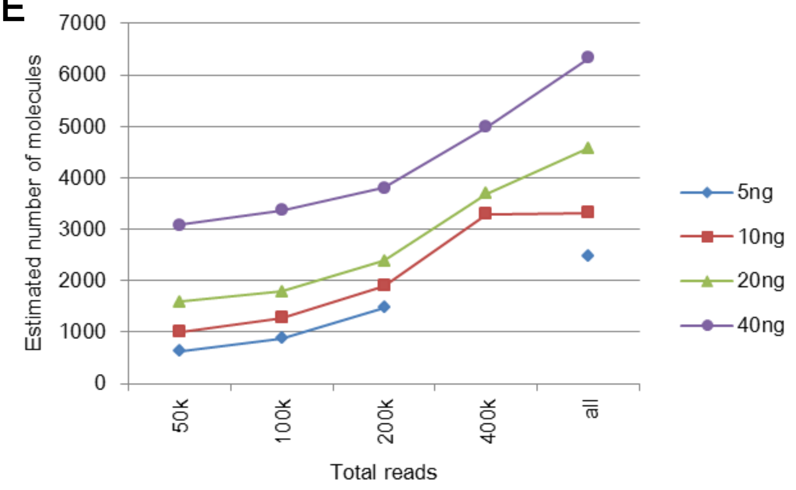

Supplementary Figure S5. Analyses of reads from the MiSeq sequencer. (**A**) Distribution of reads per barcode tag. Vertical axis: number of different barcode tags. Horizontal axis: number of reads per tag, shown as the common logarithm. (**B**) Cumulative reads. The arrow indicates the threshold for removing reads with erroneous tags. (**C**) Estimated proportion of barcode tags with or without errors. The analyzed region was TK102U. A total of 40 ng of genomic DNA was analyzed (594,719 reads). (**D**, **E**) Estimated number of target molecules after removing the erroneous barcode tags using our method (**D**) or by removing 1- or 2-read tags (**E**). Analyses were performed using reads that were randomly extracted from the entire set of reads (“5ng”: 343,932; “10ng”: 404,900; “20ng”: 548,809; “40ng”: 594,719 reads).

Supplementary Figure S6

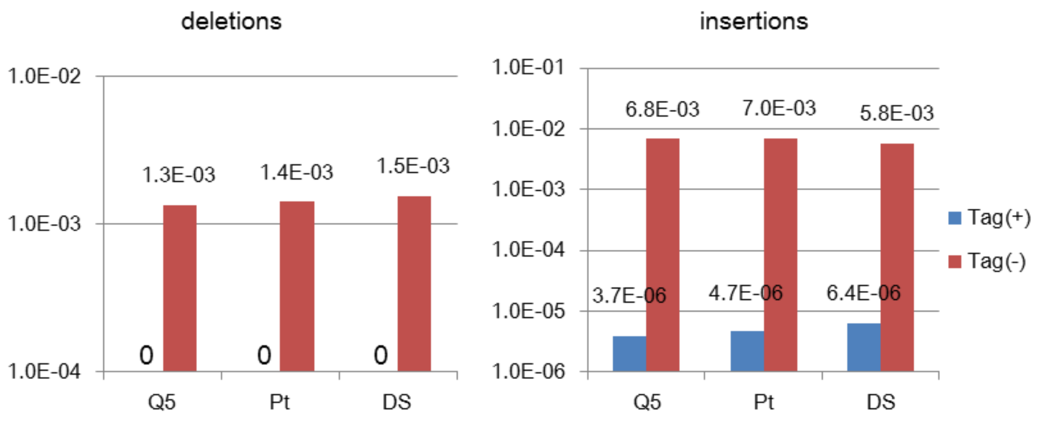

Supplementary Figure S6. Deletion and insertion errors. The error rates of target regions were analyzed with barcode tags (blue) and without (red). Sequencing libraries prepared by three different procedures were sequenced with a Proton sequencer. Q5 DNA polymerase (Q5) and Platinum Taq DNA polymerase High Fidelity kit (Pt) were used for the last amplification step of library preparation. "DS" indicates the results of double strand labeling. We analyzed 30 ng of genomic DNA (Megapool). Calculations were based on sequence data from the seven (Q5, Pt) or five (except TK102 and TK103U for DS) regions obtained with an Ion Proton sequencer.

Supplementary Table S1. Target regions

target region name	amplified region[a, b]	adaptor-attached position[a]	restriction enzyme	Set
TK102U	chr17:7577015-7577100	chr17:7577101	BcoDI (Alw26I)	Set1
TK201D	chr17:7577087-7577174	chr17:7577086	EarI	Set2
TK103U	chr17:7577493-7577558	chr17:7577559	AlwNI	Set1
TK103D	chr17:7577562-7577627	chr17:7577561	AlwNI	Set1
TK104	chr17:7578153-7578297	chr17:7578152	BcoDI (Alw26I)	Set1
TK203U	chr17:7578359-7578449	chr17:7578450	NcoI	Set2
TK203D	chr17:7578454-7578534	chr17:7578453	NcoI	Set2
KRASc12c13	chr12:25398259-25398352	chr12:25398353	NmuCI	SetKC
CTNNB1hotspots	chr3:41266074-41266158	chr3:41266159	EarI	SetKC

[a]Genomic position (hg19).

[b]Positions of primers and adaptors are not included.

Supplementary Table S2. Adaptor oligonucleotides for PGM/Proton system.

oligo name	target region[a]	sequence[b]	orientation[c]
ionBATad1S00T01	TK102U, TK104, TK201D, TK203U, TK203D, CTNNB1hotspots, KRASc12c13	biotin-CCATCTCATCCCTGCGTGTCTCCGACTCAGACAGTNNNNNNNNNNNNGTACATATTGTCGTTAGAACGCG	sense
ionBATad1S00T02	TK102U, TK104, TK201D, TK203U, TK203D, CTNNB1hotspots, KRASc12c13	biotin-CCATCTCATCCCTGCGTGTCTCCGACTCAGACGTANNNNNNNNNNNNGTACATATTGTCGTTAGAACGCG	sense
ionBATad1S00T05	TK102U, TK104, TK201D, TK203U, TK203D, CTNNB1hotspots, KRASc12c13	biotin-CCATCTCATCCCTGCGTGTCTCCGACTCAGAGCATNNNNNNNNNNNNGTACATATTGTCGTTAGAACGCG	sense
ionBATad1S00T24	TK102U, TK104, TK201D, TK203U, TK203D, CTNNB1hotspots, KRASc12c13	CCATCTCATCCCTGCGTGTCTCCGACTCAGTGACTNNNNNNNNNNNNGTACATATTGTCGTTAGAACGCG	sense
ionBATad1S00T03	CTNNB1hotspots, KRASc12c13	CCATCTCATCCCTGCGTGTCTCCGACTCAGACTACNNNNNNNNNNNNGTACATATTGTCGTTAGAACGCG	sense
ionBATad1S00T04	CTNNB1hotspots, KRASc12c13	CCATCTCATCCCTGCGTGTCTCCGACTCAGAGACGNNNNNNNNNNNNGTACATATTGTCGTTAGAACGCG	sense
ionBATad1S00T06	CTNNB1hotspots, KRASc12c13	CCATCTCATCCCTGCGTGTCTCCGACTCAGAGTGANNNNNNNNNNNNGTACATATTGTCGTTAGAACGCG	sense
ionBATad1S00T07	CTNNB1hotspots, KRASc12c13	CCATCTCATCCCTGCGTGTCTCCGACTCAGATAGCNNNNNNNNNNNNGTACATATTGTCGTTAGAACGCG	sense
ionBATad1S00T08	CTNNB1hotspots, KRASc12c13	CCATCTCATCCCTGCGTGTCTCCGACTCAGATGCTNNNNNNNNNNNNGTACATATTGTCGTTAGAACGCG	sense
ionBATad1S00T09	CTNNB1hotspots, KRASc12c13	CCATCTCATCCCTGCGTGTCTCCGACTCAGCACGTNNNNNNNNNNNNGTACATATTGTCGTTAGAACGCG	sense
ionBATad1S00T10	CTNNB1hotspots, KRASc12c13	CCATCTCATCCCTGCGTGTCTCCGACTCAGCAGACNNNNNNNNNNNNGTACATATTGTCGTTAGAACGCG	sense
ionBATad1S00T11	CTNNB1hotspots, KRASc12c13	CCATCTCATCCCTGCGTGTCTCCGACTCAGCATAGNNNNNNNNNNNNGTACATATTGTCGTTAGAACGCG	sense
ionBATad1S00T12	CTNNB1hotspots, KRASc12c13	CCATCTCATCCCTGCGTGTCTCCGACTCAGCGATANNNNNNNNNNNNGTACATATTGTCGTTAGAACGCG	sense
ionBATad1S00T14	CTNNB1hotspots, KRASc12c13	CCATCTCATCCCTGCGTGTCTCCGACTCAGCGTCGNNNNNNNNNNNNGTACATATTGTCGTTAGAACGCG	sense
ionBATad1S00T15	CTNNB1hotspots, KRASc12c13	CCATCTCATCCCTGCGTGTCTCCGACTCAGCTATGNNNNNNNNNNNNGTACATATTGTCGTTAGAACGCG	sense
ionBATad1S00T17	CTNNB1hotspots, KRASc12c13	CCATCTCATCCCTGCGTGTCTCCGACTCAGCTGCANNNNNNNNNNNNGTACATATTGTCGTTAGAACGCG	sense
ionBATad1S00T18	CTNNB1hotspots, KRASc12c13	CCATCTCATCCCTGCGTGTCTCCGACTCAGTACGANNNNNNNNNNNNGTACATATTGTCGTTAGAACGCG	sense
ionBATad1S00T19	CTNNB1hotspots, KRASc12c13	CCATCTCATCCCTGCGTGTCTCCGACTCAGTAGCGNNNNNNNNNNNNGTACATATTGTCGTTAGAACGCG	sense
ionBATad1S00T20	CTNNB1hotspots, KRASc12c13	CCATCTCATCCCTGCGTGTCTCCGACTCAGTATGCNNNNNNNNNNNNGTACATATTGTCGTTAGAACGCG	sense
ionBATad1S00T21	CTNNB1hotspots, KRASc12c13	CCATCTCATCCCTGCGTGTCTCCGACTCAGTCATCNNNNNNNNNNNNGTACATATTGTCGTTAGAACGCG	sense
ionBATad1S00T22	CTNNB1hotspots, KRASc12c13	CCATCTCATCCCTGCGTGTCTCCGACTCAGTCGAGNNNNNNNNNNNNGTACATATTGTCGTTAGAACGCG	sense
ionBATad1S00T23	CTNNB1hotspots, KRASc12c13	CCATCTCATCCCTGCGTGTCTCCGACTCAGTCTATNNNNNNNNNNNNGTACATATTGTCGTTAGAACGCG	sense
ionBATad1S00T25	CTNNB1hotspots, KRASc12c13	CCATCTCATCCCTGCGTGTCTCCGACTCAGTGCTGNNNNNNNNNNNNGTACATATTGTCGTTAGAACGCG	sense
ionBATad1S00T26	CTNNB1hotspots, KRASc12c13	CCATCTCATCCCTGCGTGTCTCCGACTCAGTGTCANNNNNNNNNNNNGTACATATTGTCGTTAGAACGCG	sense
ionBATad1S02T01	TK103U	biotin-CCATCTCATCCCTGCGTGTCTCCGACTCAGACAGTNNNNNNNNNNNNGTACATATTGTCGTTAGAACGCGTTC	sense
ionBATad1S02T02	TK103U	biotin-CCATCTCATCCCTGCGTGTCTCCGACTCAGACGTANNNNNNNNNNNNGTACATATTGTCGTTAGAACGCGTTC	sense
ionBATad1S02T05	TK103U	biotin-CCATCTCATCCCTGCGTGTCTCCGACTCAGAGCATNNNNNNNNNNNNGTACATATTGTCGTTAGAACGCGTTC	sense
ionBATad1S02T24	TK103U	CCATCTCATCCCTGCGTGTCTCCGACTCAGTGACTNNNNNNNNNNNNGTACATATTGTCGTTAGAACGCGTTC	sense
ionBATad1S01T01	TK103D	biotin-CCATCTCATCCCTGCGTGTCTCCGACTCAGACAGTNNNNNNNNNNNNGTACATATTGTCGTTAGAACGCGGAA	sense
ionBATad1S01T02	TK103D	biotin-CCATCTCATCCCTGCGTGTCTCCGACTCAGACGTANNNNNNNNNNNNGTACATATTGTCGTTAGAACGCGGAA	sense
ionBATad1S01T05	TK103D	biotin-CCATCTCATCCCTGCGTGTCTCCGACTCAGAGCATNNNNNNNNNNNNGTACATATTGTCGTTAGAACGCGGAA	sense
ionBATad1S01T24	TK103D	CCATCTCATCCCTGCGTGTCTCCGACTCAGTGACTNNNNNNNNNNNNGTACATATTGTCGTTAGAACGCGGAA	sense
ionBATad1A00T01	TK103U, TK103D	CGCGTTCTAACGACAATATGTACNNNNNNNNNNNNGTAGTCTGAGTCGGAGACACGCAGGGATGAGATGG	antisense
ionBATad1A11T01	TK102U	CCCACGCGTTCTAACGACAATATGTACNNNNNNNNNNNNGTAGTCTGAGTCGGAGACACGCAGGGATGAGATGG	antisense
ionBATad1A04T01	TK104	GGGACGCGTTCTAACGACAATATGTACNNNNNNNNNNNNGTAGTCTGAGTCGGAGACACGCAGGGATGAGATGG	antisense
ionBATad1A12T01	TK201D	AGACGCGTTCTAACGACAATATGTACNNNNNNNNNNNNCTGAGTCGGAGACACGCAGGGATGAGATGG	antisense
ionBATad1A07T01	TK203U, TK203D	CATGCGCGTTCTAACGACAATATGTACNNNNNNNNNNNNGTAGTCTGAGTCGGAGACACGCAGGGATGAGATGG	antisense
ionBATad1A15	CTNNB1hotspots	TGACGCGTTCTAACGACAATATGTACNNNNNNNNNNNNCTGAGTCGGAGACACGCAGGGATGAGATGG	antisense
ionBATad1A16	KRASc12c13	GTGACCGCGTTCTAACGACAATATGTACNNNNNNNNNNNNCTGAGTCGGAGACACGCAGGGATGAGATGG	antisense

PCR oligonucleotides

oligo name	target region[a]	sequence[d]
trP1PE_TK102U.201Up	TK102U	CCTCTCTATGGGCAGTCGGTGATCCTCAGCCCGCTTCTTGTCCTGCTTGC
trP1PE_TK103U.301Up	TK103U	CCTCTCTATGGGCAGTCGGTGATCCTCAGCGCAGGGTGGCAAGTGGCT
trP1PE_TK103D.301Dp	TK103D	CCTCTCTATGGGCAGTCGGTGATCCTCAGCGCSCACTGGCCTCATCTTG
trP1PE_TK202Dp	TK104	CCTCTCTATGGGCAGTCGGTGATCCTCAGCCAGGCCTCTGATTCCTCACKGATT

trP1PE_TK102D.201Dp2	TK201D	CCTCTCTATGGGCAGTCGGTGATCCTCAGCCCTGATTTCCTTACTGCCTCTTGCTTCT
trP1PE_TK203Up2	TK203U	CCTCTCTATGGGCAGTCGGTGATCCTCAGCAGCCCTGTCGTCTCTCCAGC
trP1PE_TK203Dp	TK203D	CCTCTCTATGGGCAGTCGGTGATCCTCAGCAGTACTCCCCTGCCCTCAACAA
CTNNB1_RU3	CTNNB1hotspots	CACTGAGTCAGACGTGTGCTACTGGCAGCAACAGTCTTAC*C*T
KRAS_RU3	KRASc12c13	CACTGAGTCAGACGTGTGCTCGTCAAGGCACTCTTGCCT*A*C
T_PCR_A	universal	CCATCTCATCCCTGCGTGTC
trP1a	universal	CCTCTCTATGGGCAGTCGGTGAT
trP1aT03	universal	CCTCTCTATGGGCAGTCGGTGATCCCGTAGTCACTGAGTCAGACGTGTGCT

[a]See Supplementary Table S1.

[b]Underlined sequences are indexes for discriminating individuals. N_{12}-mer is molecular barcode. Several 5'ends have been modified by biotins for other experiments.

[c]Index sequences for identification of individuals are not included in antisense adaptor oligonucleotides because they are not used during PCR after ligation.

[d]*, phosphorothioate linkage

Supplementary Table S3. Adaptor oligonucleotides for Illumina system.

oligo name	target region[a]	sequence[b]	orientation
ILMadS00	TK102U, TK104	ACACTCTTTCCCTACACGACGCTCTTCCGATCTAGCTABDHVBDHVBDHVBDHGTACATATTGTCGTTAGAACGCG	sense
ILMadS01	TK103U	ACACTCTTTCCCTACACGACGCTCTTCCGATCTAGCTABDHVBDHVBDHVBDHGTACATATTGTCGTTAGAACGCGTTC	sense
ILMadS02	TK103D	ACACTCTTTCCCTACACGACGCTCTTCCGATCTAGCTABDHVBDHVBDHVBDHGTACATATTGTCGTTAGAACGCGGAA	sense
ILMadA00	TK103U, TK103D	CGCGTTCTAACGACAATATGTACDHVBDHVBDHVBDHVTAGCTAGATCGGAAGAGCGTCGTGT	antisense
ILMadA11	TK102U	CCCACGCGTTCTAACGACAATATGTACDHVBDHVBDHVBDHVTAGCTAGATCGGAAGAGCGTCGTGT	antisense
ILMadA12	TK104	GGGACGCGTTCTAACGACAATATGTACDHVBDHVBDHVBDHVTAGCTAGATCGGAAGAGCGTCGTGT	antisense

PCR oligonucleotides

oligo name	target region[a]	sequence[c]
ILMGSP_TK102Up	TK102U	GTGACTGGAGTTCAGACGTGTGCTCTTCCGATCTCCGCTTCTTGTCCTGCTTGC
ILMGSP_TK103Up	TK103U	GTGACTGGAGTTCAGACGTGTGCTCTTCCGATCTGCAGGGTGGCAAGTGGCT
ILMGSP_TK103Dp	TK103D	GTGACTGGAGTTCAGACGTGTGCTCTTCCGATCTGCSCACTGGCCTCATCTTG
ILMGSP_TK104p	TK104	GTGACTGGAGTTCAGACGTGTGCTCTTCCGATCTCGAGTGGAAGGAAATTTGCGTG
ILMPCR1	universal	AATGATACGGCGACCACCGAGATCTACACTCTTTCCCTACACGACGCTCT
ILMPCRidx1	index1	CAAGCAGAAGACGGCATACGAGATCGTGATGTGACTGGAGTTCAGACGTGTGCT
ILMPCRidx2	index2	CAAGCAGAAGACGGCATACGAGATACATCGGTGACTGGAGTTCAGACGTGTGCT
ILMPCRidx3	index3	CAAGCAGAAGACGGCATACGAGATGCCTAAGTGACTGGAGTTCAGACGTGTGCT
ILMPCRidx4	index4	CAAGCAGAAGACGGCATACGAGATTGGTCAGTGACTGGAGTTCAGACGTGTGCT

[a]See Supplementary Table S1.

[b]"BDHVBDHVBDHVBDH" sequence is molecular barcode.

[c]Underlined sequences are indexes for discriminating individuals.

Supplementary Table S4. Consensus read counts of TP53 R248W mutation position.

Depth	A	C	G	T	Variant Freq. (%)	sequenced strands
1934	0	1914	0	20	1.03	one strand
2770	0	2767	0	3	0.11	double strands
2158	0	2156	0	2	0.09	double strands

Note: PCR products from Megapool and MIA CaPa-2 were mixed (ratio: Megapool : MIA CaPa-2 = 99 : 1), denatured/re-annealed and used as templates for sequencing analyses (Supplementary Figure S1). After constructed consensus of multiple reads using barcode tags, bases of R248W mutation position of TP53 were counted. Valiant frequency by both strands sequencing was 0.1%, suggesting that 90% of base changes in one strand were discriminated. "double strands" were labeled with the same barcode by replacement synthesis of the complementary strand, and sequenced (Figure 1A). For "one strand" sequencing, replacement synthesis of the complementary strand was not done.

Supplementary Table S5. Candidate restriction enzymes (FastDigest series from Thermo Scientific) for our proporsed method.

Enzyme name	Recognition sequence	Catalog number of Thermo Scientific
AatII	GACGT↓C	FD0994
Acc65I	G↓GTACC	FD0904
AgeI (BshTI)	A↓CCGGT	FD1464
AjuI	↓(7/12)GAA(N)7TTGG(11/6)↓	FD1954
Alw21I	GWGCW↓C	FD0024
Alw26I	GTCTC(1/5)↓	FD0034
AlwNI (CaiI)	CAGNNN↓CTG	FD1394
ApaI	GGGCC↓C	FD1414
ApaLI (Alw44I)	G↓TGCAC	FD0044
AscI (SgsI)	GG↓CGCGCC	FD1894
AvaI (Eco88I)	C↓YCGRG	FD0384
AvaII (Eco47I)	G↓GWCC	FD0314
BamHI	G↓GATCC	FD0054/5
BanI (BshNI)	G↓GYRCC	FD1004
BbsI (BpiI)	GAAGAC(2/6)↓	FD1014
BbvI (Lsp1109I)	GCAGC(8/12)↓	FD2074
BclI	T↓GATCA	FD0724
BglI	GCCNNNN↓NGGC	FD0074
BlpI (Bpu1102I)	GC↓TNAGC	FD0094
Bme1580I (BseSI)	GKGCM↓C	FD1444
BmtI (BspOI)	GCTAG↓C	FD2044
BplI	↓(8/13)GAG(N)5CTC(13/8)↓	FD1314
Bpu10I	CCTNAGC(-5/-2)↓	FD1184
BsaJI (BseDI)	C↓CNNGG	FD1084
BsiWI (Pfl23II)	C↓GTACG	FD0854
BsmBI (Esp3I)	CGTCTC(1/5)↓	FD0454
BsmFI (FaqI)	GGGAC(10/14)↓	FD1814
Bsp120I	G↓GGCCC	FD0134
Bsp1286I (SduI)	GDGCH↓C	FD0654
Bsp1407I	T↓GTACA	FD0933/4
BspHI (PagI)	T↓CATGA	FD1284
BspMI (BveI)	ACCTGC(4/8)↓	FD1744
BssHII (PteI)	G↓CGCGC	FD2134
BstXI	CCANNNNN↓NTGG	FD1024
Bsu36I (Eco81I)	CC↓TNAGG	FD0374
DdeI (HpyF3I)	C↓TNAG	FD1884
DraIII (AdeI)	CACNNN↓GTG	FD1234
EagI (Eco52I)	C↓GGCCG	FD0334
EarI (Eam1104I)	CTCTTC(1/4)↓	FD0234
Eco31I	GGTCTC(1/5)↓	FD0293/4
Eco91I	G↓GTNACC	FD0394
EcoO109I	RG↓GNCCY	FD0264
EcoRI	G↓AATTC	FD0274/5
FokI	GGATG(9/13)↓	FD2144
HaeII (BfoI)	RGCGT↓Y	FD2184

HgaI (CseI)	GACGC(5/10)↓	FD1904
HindIII	A↓AGCTT	FD0504/5
HinfI	G↓ANTC	FD0804
HpyF10VI	GCNNNNN↓NNGC	FD1734
Kpn2I	T↓CCGGA	FD0534
KpnI	GGTAC↓C	FD0524
MauBI	CG↓CGCGCG	FD2084
MboI	↓GATC	FD0814
MluI	A↓CGCGT	FD0564
MreI	CG↓CCGGCG	FD2024
NcoI	C↓CATGG	FD0573/4/5
NheI	G↓CTAGC	FD0973/4
NlaIII (Hin1II)	CATG↓	FD1834
NmuCI	↓GTSAC	FD1514
NotI	GC↓GGCCGC	FD0593/4/6
NsiI (Mph1103I)	ATGCA↓T	FD0734
NspI (XceI)	RCATG↓Y	FD1474
PflMI (Van91I)	CCANNNN↓NTGG	FD0714
PfoI	T↓CCNGGA	FD1754
PpuMI (Psp5II)	RG↓GWCCY	FD0764
PspFI	CCCAGC(-5/-1)↓	FD2224
PsuI	R↓GATCY	FD1554
RsrII (CpoI)	CG↓GWCCG	FD0744
SacI	GAGCT↓C	FD1133/4
SalI	G↓TCGAC	FD0644
SapI (LguI)	GCTCTTC(1/4)↓	FD1934
Sau3AI (Bsp143I)	↓GATC	FD0784
SexAI (CsiI)	A↓CCWGGT	FD2114
SfaNI (BmsI)	GCATC(5/9)↓	FD2124
SfcI (BfmI)	C↓TRYAG	FD1164
SphI (PaeI)	GCATG↓C	FD0604
StyI (Eco130I)	C↓CWWGG	FD0414
TfiI (PfeI)	G↓AWTC	FD1784
XapI	R↓AATTY	FD1383/4
XbaI	T↓CTAGA	FD0684/5
XhoI	C↓TCGAG	FD0694/5

Note: We selected FastDigest series of Thermo Scientific because they work in one universal buffer, that enables any combination of restriction enzymes in one reaction tube. All listed enzymes produce three-, four- or five-base protruding ends, and inactivate at more than 65 °C.

Supplementary Table S6. Assay data for TP53 mutation detection.

plasma sample[a]	used blood (mL)	# of molecules[b]	error bases[c]	error positions	error rate (per bp)[d]	expected error bases	P value	mutation	freq.mutation (%)	tissue	disease
before surgery	0.5	178	0	0	0.00001	0.1175	0.110842			plasma	Gastric cancer
week 1	0.5	150	3	1	0.00001	0.0990	0.000004	c.747G>C(R249S)	2.00%	plasma	Gastric cancer
month 4	1	328	0	0	0.00001	0.2165	0.194651			plasma	Gastric cancer
month 8	1	348	220	1	0.00001	0.2297	0.000000	c.747G>C(R249S)	63.22%	plasma	Gastric cancer
month 9	1	398	202	1	0.00001	0.2627	0.000000	c.747G>C(R249S)	50.75%	plasma	Gastric cancer

[a]Sampling time points after operation.

[b]Number of assayed DNA molecules was calculated from error-free barcode tags.

[c]Base changes in 66 bp-target region.

[d]Error rate of our method from Figure 4.

Supplementary Table S7. Assay data for KRAS mutation detection.

sample	used blood (mL)	used DNA (ng)	# of molecules[a]	error bases[b]	error positions	error rate (per bp)[c]	expected error bases	P value	mutation	freq.mutation (%)	tissue	disease[d]	EGFR mutation (biopsy)	age	sex
F03	---	16.1	7287	2	2	0.00001	5.2466	0.894642			leukocyte	normal	(normal control)	---	F
F04	---	11.4	4710	1	1	0.00001	3.3912	0.852156			leukocyte	normal	(normal control)	---	F
F06	---	11.6	4868	1	1	0.00001	3.5050	0.864635			leukocyte	normal	(normal control)	---	F
F07	---	20	9250	7	6	0.00001	6.6600	0.350768			leukocyte	normal	(normal control)	---	F
F08	---	8.6	3074	4	4	0.00001	2.2133	0.073940			leukocyte	normal	(normal control)	---	F
F10	---	10	4026	6	4	0.00001	2.8987	0.028659			leukocyte	normal	(normal control)	---	F
F12	---	23	8661	3	3	0.00001	6.2359	0.868640			leukocyte	normal	(normal control)	---	F
M01	---	10.5	4901	1	1	0.00001	3.5287	0.867116			leukocyte	normal	(normal control)	---	M
M02	---	7	3587	4	4	0.00001	2.5826	0.120133			leukocyte	normal	(normal control)	---	M
M06	---	12.2	3127	0	0	0.00001	2.2514	0.894752			leukocyte	normal	(normal control)	---	M
M10	---	10	3125	1	1	0.00001	2.2500	0.657453			leukocyte	normal	(normal control)	---	M
DP01	1	---	931	1	1	0.00001	0.6703	0.145556			plasma	normal	(normal control)	---	---
DP02	1	---	719	1	1	0.00001	0.5177	0.095612			plasma	normal	(normal control)	---	---
DP07	1	---	117	0	0	0.00001	0.0842	0.080789			plasma	normal	(normal control)	---	---
DP11	1	---	425	1	1	0.00001	0.3060	0.038279			plasma	normal	(normal control)	---	---
DP01_2	1	---	113	0	0	0.00001	0.0814	0.078138			plasma	normal	(normal control)	---	---
DP02_2	1	---	947	0	0	0.00001	0.6818	0.494314			plasma	normal	(normal control)	---	---
DP07_2	1	---	502	0	0	0.00001	0.3614	0.303328			plasma	normal	(normal control)	---	---
K017	0.3	---	840	54	2	0.00001	0.6048	0.000000	c.34G>T(G12C)	6.43%	plasma	NSCLC (adenocarcinoma)	wild	81	M
K117	0.3	---	473	12	1	0.00001	0.3406	0.000000	c.34G>T(G12C)	2.54%	plasma	NSCLC (adenocarcinoma)	wild	73	F
K121	0.5	---	1412	9	3	0.00001	1.0166	0.000000	c.34G>T(G12C)	0.64%	plasma	NSCLC (adenocarcinoma)	wild	56	M
K143	0.5	---	685	31	1	0.00001	0.4932	0.000000	c.35G>A(G12D)	4.53%	plasma	NSCLC (adenocarcinoma)	wild	65	M
K207	0.7	---	1064	203	1	0.00001	0.7661	0.000000	c.35G>C(G12A)	19.08%	plasma	NSCLC (adenocarcinoma)	wild	55	F
K277	0.5	---	452	0	0	0.00001	0.3254	0.277790			plasma	NSCLC (adenocarcinoma)	wild	71	M
K319	0.5	---	1302	305	1	0.00001	0.9374	0.000000	c.34G>T(G12C)	23.43%	plasma	NSCLC (adenocarcinoma)	wild	61	M
K324	0.5	---	3476	1	1	0.00001	2.5027	0.713260			plasma	NSCLC (adenocarcinoma)	wild	69	M
K367	0.7	---	254	0	0	0.00001	0.1829	0.167132			plasma	NSCLC (adenocarcinoma)	wild	69	F
K373	0.5	---	909	0	0	0.00001	0.6545	0.480288			plasma	NSCLC (adenocarcinoma)	wild	72	F
K381	0.3	---	132	16	2	0.00001	0.0950	0.000000	c.35G>T(G12V)	12.12%	plasma	NSCLC (adenocarcinoma)	wild	59	M
K407	0.5	---	9134	3128	4	0.00001	6.5765	0.000000	c.34G>T(G12C)	34.25%	plasma	NSCLC (adenocarcinoma)	wild	71	M
K439	0.7	---	104	0	0	0.00001	0.0749	0.072145			plasma	NSCLC (adenocarcinoma)	wild	79	M
K519	0.5	---	369	0	0	0.00001	0.2657	0.233316			plasma	NSCLC (adenocarcinoma)	wild	73	M
K545	0.5	---	264	0	0	0.00001	0.1901	0.173107			plasma	NSCLC (adenocarcinoma)	exon19del	68	F
K582	0.5	---	179	1	1	0.00001	0.1289	0.007625			plasma	NSCLC (adenocarcinoma)	exon19del	71	M
K649	0.7	---	201	0	0	0.00001	0.1447	0.134735			plasma	NSCLC (adenocarcinoma)	L858R	56	F
K650	0.5	---	163	0	0	0.00001	0.1174	0.110735			plasma	NSCLC (adenocarcinoma)	wild	75	F
K655	0.7	---	442	0	0	0.00001	0.3182	0.272572			plasma	NSCLC (adenocarcinoma)	exon19del	63	M
K661	0.5	---	348	0	0	0.00001	0.2506	0.221635			plasma	NSCLC (adenocarcinoma)	L858R	64	M
K683	0.5	---	257	0	0	0.00001	0.1850	0.168929			plasma	NSCLC (adenocarcinoma)	exon19del	---	F
K684	0.5	---	118	0	0	0.00001	0.0850	0.081451			plasma	NSCLC (adenocarcinoma)	L858R	66	F
K686	0.5	---	85	0	0	0.00001	0.0612	0.059365			plasma	NSCLC (adenocarcinoma)	wild	72	M
K689	0.5	---	195	0	0	0.00001	0.1404	0.130989			plasma	NSCLC (adenocarcinoma)	exon19del	85	M

K706	0.5	---	491	1	1	0.00001	0.3535	0.049542			plasma	NSCLC (adenocarcinoma)	wild	78	F
K708	0.5	---	159	5	1	0.00001	0.1145	0.000000	c.9A>G(E3E)	3.14%	plasma	NSCLC (adenocarcinoma)	L858R	58	M
K717	0.7	---	191	0	0	0.00001	0.1375	0.128483			plasma	NSCLC (adenocarcinoma)	exon19del	47	M
K758	1	---	595	1	1	0.00001	0.4284	0.069325			plasma	NSCLC (adenocarcinoma)	wild	67	M
K762	1	---	415	0	0	0.00001	0.2988	0.258292			plasma	NSCLC (adenocarcinoma)	L858R	57	F
K763	1	---	277	0	0	0.00001	0.1994	0.180811			plasma	NSCLC (adenocarcinoma)	wild	59	M

[a]Number of assayed DNA molecules was calculated from error-free barcode tags.

[b]Base changes in 72 bp-target region (excluded 1 base position, where is an error-hotspot due to homopolymeric region).

[c]Error rate of our method from Figure 4.

[d]NSCLC: Non-small cell lung cancer

Supplementary Table S8. Assay data for CTNNB1 mutation detection.

sample	used blood (mL)	used DNA (ng)	# of molecules[a]	error bases[b]	error positions	error rate (per bp)[c]	expected error bases	P value	mutation	freq.mutation (%)	tissue	disease[d]	EGFR mutation (biopsy)	age	sex
F03	---	16.1	6633	2	2	0.00001	4.1788	0.786941			leukocyte	normal	(normal control)	---	F
F04	---	11.4	4782	3	3	0.00001	3.0127	0.355604			leukocyte	normal	(normal control)	---	F
F06	---	11.6	4821	2	2	0.00001	3.0372	0.585099			leukocyte	normal	(normal control)	---	F
F07	---	20	8727	11	9	0.00001	5.4980	0.010960			leukocyte	normal	(normal control)	---	F
F08	---	8.6	2994	3	3	0.00001	1.8862	0.122951			leukocyte	normal	(normal control)	---	F
F10	---	10	4194	6	6	0.00001	2.6422	0.018553			leukocyte	normal	(normal control)	---	F
F12	---	23	8658	10	6	0.00001	5.4545	0.023978			leukocyte	normal	(normal control)	---	F
M01	---	10.5	4686	2	2	0.00001	2.9522	0.566011			leukocyte	normal	(normal control)	---	M
M02	---	7	3638	1	1	0.00001	2.2919	0.667283			leukocyte	normal	(normal control)	---	M
M06	---	12.2	3177	1	1	0.00001	2.0015	0.594403			leukocyte	normal	(normal control)	---	M
M10	---	10	3403	8	8	0.00001	2.1439	0.000391	8errors/8sites		leukocyte	normal	(normal control)	---	M
DP01	1	---	652	0	0	0.00001	0.4108	0.336854			plasma	normal	(normal control)	---	---
DP02	1	---	756	0	0	0.00001	0.4763	0.378910			plasma	normal	(normal control)	---	---
DP07	1	---	190	0	0	0.00001	0.1197	0.112813			plasma	normal	(normal control)	---	---
DP11	1	---	334	0	0	0.00001	0.2104	0.189756			plasma	normal	(normal control)	---	---
DP01_2	1	---	97	0	0	0.00001	0.0611	0.059280			plasma	normal	(normal control)	---	---
DP02_2	1	---	800	0	0	0.00001	0.5040	0.395891			plasma	normal	(normal control)	---	---
DP07_2	1	---	389	0	0	0.00001	0.2451	0.217350			plasma	normal	(normal control)	---	---
K017	0.3	---	554	4	1	0.00001	0.3490	0.000032	c.114T>C(G38G)	0.72%	plasma	NSCLC (adenocarcinoma)	wild	81	M
K117	0.3	---	360	0	0	0.00001	0.2268	0.202920			plasma	NSCLC (adenocarcinoma)	wild	73	F
K121	0.5	---	1329	2	2	0.00001	0.8373	0.052930			plasma	NSCLC (adenocarcinoma)	wild	56	M
K143	0.5	---	535	0	0	0.00001	0.3371	0.286127			plasma	NSCLC (adenocarcinoma)	wild	65	M
K207	0.7	---	788	41	1	0.00001	0.4964	0.000000	c.110C>G(S37C)	5.20%	plasma	NSCLC (adenocarcinoma)	wild	55	F
K277	0.5	---	255	0	0	0.00001	0.1607	0.148410			plasma	NSCLC (adenocarcinoma)	wild	71	M
K319	0.5	---	1196	0	0	0.00001	0.7535	0.529274			plasma	NSCLC (adenocarcinoma)	wild	61	M
K324	0.5	---	2612	3	3	0.00001	1.6456	0.085217			plasma	NSCLC (adenocarcinoma)	wild	69	M
K367	0.7	---	139	0	0	0.00001	0.0876	0.083845			plasma	NSCLC (adenocarcinoma)	wild	69	F
K373	0.5	---	801	0	0	0.00001	0.5046	0.396271			plasma	NSCLC (adenocarcinoma)	wild	72	F
K381	0.3	---	140	0	0	0.00001	0.0882	0.084422			plasma	NSCLC (adenocarcinoma)	wild	59	M
K407	0.5	---	7477	6	6	0.00001	4.7105	0.196828			plasma	NSCLC (adenocarcinoma)	wild	71	M
K439	0.7	---	77	0	0	0.00001	0.0485	0.047352			plasma	NSCLC (adenocarcinoma)	wild	79	M
K519	0.5	---	136	0	0	0.00001	0.0857	0.082112			plasma	NSCLC (adenocarcinoma)	wild	73	M
K545	0.5	---	269	0	0	0.00001	0.1695	0.155888			plasma	NSCLC (adenocarcinoma)	exon19del	68	F
K582	0.5	---	159	0	0	0.00001	0.1002	0.095316			plasma	NSCLC (adenocarcinoma)	exon19del	71	M
K649	0.7	---	193	0	0	0.00001	0.1216	0.114489			plasma	NSCLC (adenocarcinoma)	L858R	56	F
K650	0.5	---	149	0	0	0.00001	0.0939	0.089599			plasma	NSCLC (adenocarcinoma)	wild	75	F
K655	0.7	---	274	0	0	0.00001	0.1726	0.158543			plasma	NSCLC (adenocarcinoma)	exon19del	63	M
K661	0.5	---	255	0	0	0.00001	0.1607	0.148410			plasma	NSCLC (adenocarcinoma)	L858R	64	M
K683	0.5	---	247	1	1	0.00001	0.1556	0.010922			plasma	NSCLC (adenocarcinoma)	exon19del	---	F
K684	0.5	---	170	0	0	0.00001	0.1071	0.101564			plasma	NSCLC (adenocarcinoma)	L858R	66	F
K686	0.5	---	137	1	1	0.00001	0.0863	0.003517			plasma	NSCLC (adenocarcinoma)	wild	72	M
K689	0.5	---	231	0	0	0.00001	0.1455	0.135436			plasma	NSCLC (adenocarcinoma)	exon19del	85	M

K706	0.5	---	333	0	0	0.00001	0.2098	0.189246		plasma	NSCLC (adenocarcinoma)	wild	78	F
K708	0.5	---	156	0	0	0.00001	0.0983	0.093605		plasma	NSCLC (adenocarcinoma)	L858R	58	M
K717	0.7	---	115	0	0	0.00001	0.0725	0.069888		plasma	NSCLC (adenocarcinoma)	exon19del	47	M
K758	1	---	545	1	1	0.00001	0.3434	0.047041		plasma	NSCLC (adenocarcinoma)	wild	67	M
K762	1	---	590	0	0	0.00001	0.3717	0.310439		plasma	NSCLC (adenocarcinoma)	L858R	57	F
K763	1	---	312	0	0	0.00001	0.1966	0.178448		plasma	NSCLC (adenocarcinoma)	wild	59	M

[a]Number of assayed DNA molecules was calculated from error-free barcode tags.

[b]Base changes in 63 bp-target region.

[c]Error rate of our method from Figure 4.

[d]NSCLC: Non-small cell lung cancer
